# Epigenetic Modifications in Acute Myeloid Leukemia: Prognosis, Treatment, and Heterogeneity

**DOI:** 10.3389/fgene.2019.00133

**Published:** 2019-03-01

**Authors:** Samantha L. Goldman, Ciaran Hassan, Mihir Khunte, Arielle Soldatenko, Yunji Jong, Ebrahim Afshinnekoo, Christopher E. Mason

**Affiliations:** ^1^ Department of Physiology and Biophysics, Weill Cornell Medicine, New York, NY, United States; ^2^ The HRH Prince Alwaleed Bin Talal Bin Abdulaziz Alsaud Institute for Computational Biomedicine, Weill Cornell Medicine, New York, NY, United States; ^3^ University of Maryland, College Park, MD, United States; ^4^ Yale College, New Haven, CT, United States; ^5^ The WorldQuant Initiative for Quantitative Prediction, Weill Cornell Medicine, New York, NY, United States; ^6^ The Feil Family Brain and Mind Research Institute, Weill Cornell Medicine, New York, NY, United States

**Keywords:** acute myeloid leukemia, AML, epigenetics, heterogeneity, epialleles

## Abstract

Leukemia, specifically acute myeloid leukemia (AML), is a common malignancy that can be differentiated into multiple subtypes based on leukemogenic history and etiology. Although genetic aberrations, particularly cytogenetic abnormalities and mutations in known oncogenes, play an integral role in AML development, epigenetic processes have been shown as a significant and sometimes independent dynamic in AML pathophysiology. Here, we summarize how tumors evolve and describe AML through an epigenetic lens, including discussions on recent discoveries that include prognostics from epialleles, changes in RNA function for hematopoietic stem cells and the epitranscriptome, and novel epigenetic treatment options. We further describe the limitations of treatment in the context of the high degree of heterogeneity that characterizes acute myeloid leukemia.

## Introduction

Acute myeloid leukemia is a common, heterogeneous, and aggressive hematopoietic malignancy (Hasserjian, 2013; Saultz and Garzon, 2016; De Kouchkovsky and Abdul-Hay, 2016), which can be differentiated into subtypes based on the nature of the (often cytogenetic) initiating leukemogenic event (Lazarus and Litzow, 2012; Hou et al., 2013; Hasserjian, 2013; Saultz and Garzon, 2016). There are two common classification systems for the characterization of acute myeloid leukemia subtypes: the French-American-British (FAB) system, which morphologically distinguishes leukemias by progenitor cell type and cell maturation; and the World Health Organization (WHO) system, which distinguishes leukemias by their characteristic cytogenetic or genetic abnormalities (Saultz and Garzon, 2016). Though there are many acute myeloid leukemia subtypes driven by epigenetic dysregulation, a brief survey of some common AML subtypes and relevant epigenetic context is given below.

## t(15;17)

The t(15;17) chromosomal abnormality is a translocation that fuses the promyelocytic locus (*PML*) gene, which encodes an eponymous tumor-suppressor protein (Salomoni and Pandolfi, 2002) on chromosome 15, with the retinoic acid receptor-α (*RARA*) gene on chromosome 17, resulting in the *PML-RARA* fusion gene (Fu et al., 1995). Both *PML* and *RARA* genes are known to be involved in regular cellular functions: *PML* is involved in the regulation of cellular proliferation (Salomoni and Pandolfi, 2002), and *RARa* is integral in myeloid differentiation and regular hematopoietic development (Melnick and Licht, 1999). Thus, the disruption of both can contribute to a hematopoietic malignancy characterized by enhanced cellular proliferation, such as what is seen in acute myeloid leukemia. Indeed, it has been shown that the introduction of *PML*-*RARa* into myeloid cells (a *TF-1* leukemia cell line) inhibits regularly induced cellular apoptosis (Fu et al., 1995) and blocks myeloid differentiation (Grignani et al., 1993).

Though the t(15;17) translocation is reciprocal – it generates both *PML*-*RARa* and *RARa-PML* gene fusions (Gallagher et al., 1995), with both being implicated in leukemogenesis (Lafage-Pochitaloff et al., 1995; Richter et al., 2016) – the *PML-RARa* product seems to be of primary importance (Gallagher et al., 1995). The *PML-RARa* fusion is pathognomonic of acute promyelocytic leukemia (Fu et al., 1995), a subtype of acute myeloid leukemia characterized by a high response rate to all-trans retinoic acid (ATRA)/arsenic trioxide (ATO) therapy and a comparatively favorable prognosis (de Thé and Chen, 2010; Coombs et al., 2015).

The *PML-RARa* product works in tandem with DNA methyltransferases (DNMTs) to induce hypermethylation in *PML-RARa* targets, particularly *RARβ* (Di Croce, 2002). Indeed, the *PML-RARa* product seems to be a necessary component for the development of a hypermethylated phenotype. For instance, mice possessing DNMT3a1 but lacking the *PML-RARa* product did not display a hypermethylated phenotype, while leukemic mice possessing both the *PML-RARa* product and DNMT3a1 presented with the hypermethylated phenotype (Subramanyam et al., 2010). Notably, retinoic acid reverses this hypermethylated phenotype (Di Croce, 2002), indicating that utility of ATRA treatment for acute promyelocytic leukemia functions in part due to the epigenetic nature of its mechanism of action.

## t(8;21)

The t(8;21) chromosomal translocation fuses the *RUNX1* and *RUNX1T1* genes (also known as AML1-ETO) (Nishii et al., 2003; Krauth et al., 2014). The *RUNX1* gene encodes runt-related transcription factor 1 (or acute myeloid leukemia factor 1/AML1) and plays a regulatory role in hematopoietic development (Okuda et al., 2001; Wichmann et al., 2015), including in the generation and differentiation of hematopoietic stem cells (Asou, 2003). Mutational aberrations in the *RUNX1* gene have been shown to reduce the expression of CCAAT enhancer-binding protein alpha (*CEBPA*), a transcription factor involved in cell cycle regulation and myeloid differentiation (Grossmann et al., 2012). The AML1-ETO protein generated by the t(8;21) fusion gene downregulates *CEBPA* expression (Pabst, 2001), playing a role in the development and pathology of acute myeloid leukemia (Yan et al., 2004; Pabst and Mueller, 2009).

In the AML1-ETO fusion protein, the ETO domain aids in the recruitment of histone deacetylases (Yan et al., 2004; Liu et al., 2007), epigenetically driving the arrest of myeloid differentiation in t(8;21)-positive leukemia (Liu et al., 2007; Wichmann et al., 2015; Loke et al., 2017) and contributing to leukemogenesis (Liu et al., 2007; Loke et al., 2017). Indeed, upon the selective removal of AML1-ETO, previously blocked myeloid differentiation is induced and leukemic proliferation halts (Loke et al., 2017).

The leukemogenic ability of the AML1-ETO product may be partly dependent on post-translational lysine acetylation of the fusion protein. Wang et al. found median survival in leukemic mouse models was increased *via* inhibition of the lysine acetyltransferase p300 (Wang et al., 2011), which reduced Lys^43^ acetylation levels in AML1-ETO9a, a splice isoform of AML1-ETO (Zhang et al., 2007; Link et al., 2016). Though p300 knockdown leads to decreased acetylation, the therapeutic effects of p300 knockdown could be due to effects largely unrelated to AML1-ETO9a acetylation, indicating p300 may be a broader therapeutic target (Wang et al., 2011).

Similarly, post-translational arginine methylation of the AML1-ETO9a protein *via* protein arginine methyltransferase 1 (PRMT1) may affect leukemic potential (Shia et al., 2012). Though PRMT1 methylates AML1-ETO9a and PRMT1 knockdown reduces leukemic proliferation, it is important to note that PRMT1 weakly methylates the AML1-ETO9a arginine (Shia et al., 2012). Thus, similar to the case of p300 knockdown, it is unclear whether leukemic proliferation is reduced by virtue of lesser arginine methylation or by virtue of inhibiting additional PRMT1-mediated interactions – for example, the recruitment of PRMT1 by AML1-ETO9a to methylate histone H4 to upregulate transcription (Shia et al., 2012).

The bulk of the therapeutic potential of PRMT1 inhibition, then, may be derived less from the consequent reduction of arginine methylation and more from the inhibition of interaction of PRMT1 with additional substrates, indicating the role of PRMT1 as a broader therapeutic target (Shia et al., 2012). Indeed, many PRMTs have been indicated as potential therapeutic targets for AML. For instance, PRMT4 inhibition enhances myeloid differentiation and inhibits myeloid proliferation (Vu et al., 2013), while PRMT5 inhibition promotes myeloid differentiation (Kaushik et al., 2017).

More recently, it has been shown that the AML1-ETO induced silencing of brain acid-soluble protein 1 (BASP1) contributes to the leukemic phenotype: AML1-ETO product-mediated recruitment of a DNA methyltransferase to the BASP1-promoter region leads to promoter methylation and ultimate silencing of BASP1, the expression of which regulates the cell cycle and arrests the cellular proliferation (Zhou et al., 2018).

Though acute myeloid leukemias characterized by the t(8;21) cytogenetic abnormality show generally favorable response to conventional chemotherapy and have a relatively favorable prognosis (Nishii et al., 2003; Krauth et al., 2014), accompanying aberrations in other respects can impact prognosis. For instance, mutations related to tyrosine-protein kinase kit, encoded by the *KIT* gene, can confer chemoresistance (Wichmann et al., 2015) and negatively impact the overall survival prognosis in t(8;21)-positive acute myeloid leukemias (Krauth et al., 2014).

## inv(16)/t(16;16)

The inv(16) chromosomal inversion is an inversion that fuses the core-binding factor subunit beta (*CBFB*) gene to the myosin heavy chain 11 (*MYH11*) gene, producing a *CBFB-MYH11* fusion gene and resulting in the expression of a CBFB-SMMHC fusion protein (Eghtedar et al., 2012; Richter et al., 2016). While the CBFB-SMMHC fusion protein plays a critical role in leukemogenesis, it does not independently initiate the disease, since CBFB-SMMHC initiated leukemogenesis has been shown in mouse models to require the cooperation of additional gene partners (Castilla et al., 1999; Castilla et al., 2004).

Unlike the t(15;17) reciprocal translocation, in which both the *PML-RARa* product and the reciprocal *RARa-PML* product are implicated in leukemogenesis (Lafage-Pochitaloff et al., 1995), the reciprocal product *MYH11-CBFB* for inv(16) is thought to be largely irrelevant to leukemogenesis (Marlton et al., 1995; Richter et al., 2016), with the lion’s share of leukemogenic activity coming from the *CBFB-MYH11* transcript (Marlton et al., 1995). Functionally, the inv(16) cytogenetic inversion produces the same fusion product as the t(16;16) cytogenetic translocation (Eghtedar et al., 2012), and it has been shown that there is no statistically significant survival difference between t(16;16) cases and inv(16) cases of acute myeloid leukemia (Larson et al., 1986). Furthermore, AMLs characterized by inv(16) and the related t(16;16) show favorable prognosis (Larson et al., 1986; Delaunay, 2003; Eghtedar et al., 2012; Richter et al., 2016).

A driver of the leukemic phenotype in inv(16) leukemia is the histone deacetylase 8 (HDAC8)-mediated deacetylation of the p53 protein (Qi et al., 2015). HDAC8 expression is significantly enriched in inv(16) + AML cells compared to hematopoietic controls, and HDAC8 inhibition reduced inv(16) + AML cell viability, reducing cellular proliferation and inducing apoptosis in the AML cells while leaving control hematopoietic cells unaffected (Qi et al., 2015).

It seems that comparatively little work has been done to elucidate the mechanisms of epigenetic dysregulation in the inv(16) subtype. However, it has been shown that inv(16) AMLs tend to feature a hypomethylated phenotype relative to non-inv(16) AMLs, with the bulk of this hypomethylation occurring in the meningioma 1 (*MN1*) oncogene (Larmonie et al., 2017).

## Epigenetic Discriminants within Acute Myeloid Leukemia

Epigenetic modifications comprise a class of heritable, non-genetic changes in gene expression, which commonly include DNA methylation, histone modification, and chromatin remodeling (Allis and Jenuwein, 2016). In healthy hematopoietic stem cells, epigenetic processes play a critical role in cell differentiation and hematopoiesis (Cullen et al., 2014; Kramer and Challen, 2017). Moreover, aberrations in epigenetic processes that disrupt normal hematopoiesis are implicated as contributory to the development of healthy hematopoietic cells into their leukemogenic counterparts (Hu and Shilatifard, 2016).

Indeed, epigenetics has been implicated as relevant to acute myeloid leukemia pathophysiology, as well as in colon cancer and in the CpG Island Methylator Phenotype (CIM) in glioblastoma (Toyota et al., 1999; Plass et al., 2008; Turcan et al., 2012). Further, Welch et al. have demonstrated that the mutational distribution of hematopoietic stem cells collected from healthy subjects is largely indifferent from the mutational distribution of the genomes of cells collected from AML samples. Some of the mutational burden observed in AML genomes is seemingly a result of random mutations that necessarily accumulate as a healthy progenitor cell ages, a process referred to as clonal hematopoiesis of indeterminate potential (CHIP) (Busque et al., 2012; Steensma et al., 2015). Upon the initiation of a leukemogenic event, the pre-existing mutations naturally accumulated by age in the progenitor cell are propagated into the now-cancerous genome (Welch et al., 2012).

AML genomes additionally tend to be less mutated relative to the genomes of other cancers (The Cancer Genome Atlas Research Network, 2013), with similar mutational distributions pre-relapse and post-relapse (Li et al., 2016a,b); furthermore, of these mutations, many tend to occur in genes associated with DNA methylation and epigenetic regulation, including *DNMT3A*, *TET1/2*, and *IDH1/2* (Guillamot et al., 2016). These results suggest a relative independence between the progression of acute myeloid leukemia and the strictly genetic landscape of the disease, further indicating an increased importance for understanding AML through a non-genetic lens (Hassan et al., 2017), with a particular focus on DNA methylation and other epigenetic modalities.

A recent five-stage, relapsed AML study revealed several aspects of this genetic and epigenetic independence, specifically using an analysis of cytosine methylation at a series of consecutive CpG dinucleotides, with each of 16 possible methylation patterns at these loci termed an “epiallele” (Li et al., 2016a,b). The overall shift in these methylation patterns (the degree of “epigenetic shift”) across the genome of an AML specimen was quantified (as an entropy shift – delta Boltzmann entropy), with the magnitude of the overall methylation shift across a genome exhibiting prognostic relevance (Li et al., 2016a,b). An analysis of a cohort of AML patients split by median epigenetic shift demonstrated that patients with a high magnitude of epigenetic shift at diagnosis relapsed more quickly than did patients with a low magnitude of epigenetic shift (*p* = 0.0396, Mantel-Cox log rank test). The prognostic significance of epigenetic shift was preserved (*p* = 0.024, Cox proportional hazards regression model) in a subsequent multivariate analysis controlling for additional clinical variables including age, white blood cell count at diagnosis, and gender. None of these additional clinical variables was singularly significantly associated with time-to-relapse in the studied AML cohort, and Li et al. additionally found no significant association between the degree of somatic mutations and time-to-relapse (Li et al., 2016a,b).

Methylation signatures are further discriminative of acute myeloid leukemia subtypes. Figueroa et al. compiled a set of methylation profiles (50,000 CpG dinucleotides across ~14,000 unique gene loci) of blast cells collected from 344 AML subjects, clustering them *via* an unsupervised hierarchical method into 16 distinct clusters (Figueroa et al., 2010), each characterized by a unique epigenetic signature. Three of the generated epigenetic signature-differentiated clusters featured strong overlap with three established cytogenetic classes of acute myeloid leukemia commonly designated as having a relatively favorable prognosis: t(8;21) (Nishii et al., 2003); inv(16)/t(16;16) (Delaunay, 2003); and t(15;17) (Lavau and Dejean, 1994; Figueroa et al., 2010; Iland and Seymour, 2013). These subclasses have already shown some significance for stratifying treatment options in combination therapy, based on TET and IDH mutations (Shih et al., 2017).

Interestingly, cluster 3, an epigenetically distinct cluster that features strong overlap with the established t(8;21) cytogenetic class, also contains individual leukemia cases that do not feature the cytogenetic abnormality characteristic of their cluster’s class. Though cluster 3 is defined as a t(8;21) cluster due to being significantly enriched (*p* < 1.85E-25, Fisher’s exact test) with t(8;21) positive patients, the epigenetic cluster contains individual cases that are not positive for the cluster’s characteristic t(8;21) abnormality. Despite the cytogenetic difference, there is no significant difference (*p* = 0.83, log-rank test) in survival between cluster 3 patients with t(8;21) and cluster 3 patients without t(8;21), indicating that the underlying methylation signature is the primarily discriminative feature that predicts survival (Figueroa et al., 2010). An additional five of the generated clusters are characterized *only* by their unique epigenetic signatures – these clusters are not significantly enriched with or differentiated by other molecular, cytogenetic, or clinical factors known to be associated with acute myeloid leukemia. Figueroa et al. further report an epigenetic signature consisting of 45 genes, many hypermethylated, that feature consistent dysregulation across multiple AML subtypes and a supermajority of the AML cases studied. Additionally, the authors report a 16-gene methylation-based classifier predictive of AML-patient survival (*p* < 0.001) (Figueroa et al., 2010).

More recently, Singh et al. have identified DNA methylation and histone acetylation signatures discriminative of high-risk acute promyelocytic leukemia (APL), which, as described above, is often induced by a *PML-RARa* fusion oncogene (Lavau and Dejean, 1994; Figueroa et al., 2010; Iland and Seymour, 2013 Singh et al., 2018). Specifically, Singh et al. analyzed both the genomes and the epigenomes of two “high-risk” APL patients, characterized by a resistance to the all-trans-retinoic acid (ATRA) therapy commonly used to successfully treat APL (de Thé and Chen, 2010; Coombs et al., 2015; Singh et al., 2018) in comparison with ATRA-sensitive “lower-risk” APL patients. While there were no significantly discriminative genetic features separating the two risk groups in comparison with lower-risk APL cases, high-risk APL was characterized by a set of distinct epigenetic signatures. Through supervised clustering, Singh et al. identified a set of differentially methylated CpG sites between the high-risk APL and the low-risk APL groups, with a 23 CpG signature capable of differentiating high-risk APL cases (Singh et al., 2018).

A separate unsupervised hierarchical clustering analysis by Schoofs et al. found that, compared to healthy controls, AML genomes tend to be characterized by increased methylation variability (à la the “epigenetic shift” described by Li et al.). (Li et al., 2016a,b) and tend to feature DNA hypermethylation (with hypomethylation occurring at chromosomal ends) (Schoofs et al., 2012). Interestingly, Schoofs et al. noted that *PML*-*RARa-*binding sites were generally lacking of aberrant methylation signatures, despite the *PML*-*RARa* fusion being the hallmark initiating cytogenetic event for acute promyelocytic leukemia (Schoofs et al., 2012; De Braekeleer et al., 2014; Singh et al., 2018). Singh et al., however, found a distinct hyperacetylation of *PML*-*RARa-*binding sites in high-risk APL patients compared to low-risk APL samples, both untreated and post-treatment with all-trans-retinoic acid (Singh et al., 2018).

Finally, Gebhard et al. have recently differentiated CG-rich classes of AML through methylation profiling, which revealed that AML could be divided into two subgroups based on the likely mechanism of methylation. The first group was associated with repression of polycomb group (PcG) proteins, a class of proteins which contributes to epigenetic silencing of genes *via* chromatin remodeling. Methylation of these genes was found in all of the AML samples analyzed. The second group was comprised of highly heterogeneous hypo- and hyper-methylated samples. These methylation patterns demonstrate the true complexity of methylation in AML (Gebhard et al., 2018).

## Approaches to Epigenetic Therapy

As the role of DNA methylation, histone acetylation, and related epigenetic events is, as described above, relevant to AML pathophysiology, the use of epigenetically focused therapies for AML is an area of active research. Several treatments targeting the epigenetic mechanisms and heterogeneity of AML are currently being explored.

## Histone Methyltransferase Inhibitors

Histone methyltransferases can regulate gene transcription through the addition of a methyl group to a lysine (Murray, 1964) or arginine residue (Byvoet et al., 1972). Depending on the location of histone methylation and the number of methyl groups added to the residue, this process can lead to either gene transcription (Bernstein et al., 2002) or gene repression (Snowden et al., 2002). Many histone methyltransferases belong to the SET domain methyltransferase protein superfamily (Dillon et al., 2005), which transfer a methyl group from S-adenosyl-L-methionine (SAM) to the lysine residue, leaving a methylated lysine residue on the histone protein and S-adeno-L-homocysteine (SAH) as a byproduct of the reaction (Dillon et al., 2005). A similar process is also implicated in RNA methylation (Saletore et al., 2012), which has also recently been implicated in controlling stem cell differentiation in AML (Vu et al., 2017) and which can now be detected with single-molecule methods to create epialleles of phased RNA modifications on RNA (Novoa et al., 2017), just as we have described here for DNA.

Enhancer of Zest Homologue 2 (EZH2) is a histone lysine methyltransferase that catalyzes the Polycomb Repressive Complex 2 (PRC2) (Cao et al., 2002). PRC2 is involved in regular cellular division, and upregulation of its catalytic subunit, EZH2, has been shown to be a marker for prostate (Xu et al., 2012) and breast cancer (Kleer et al., 2003). Mutations in EZH2 can also change its substrate specificity. While the wild-type version of EZH2 normally leads to the conversion of H3K27 from mono-methylated to di-methylated, mutations in EZH2 further converts the H3K27 from di-methylated to tri-methylated. These mutations have been implicated in various types of lymphoma (Wigle et al., 2011).

Singh et al. explored the inhibition of EZH2 with a novel EZH2-inhibitor, MC2884. In an *ex vivo* analysis of high-risk acute promyelocytic leukemia (APL), Singh et al. found that the administration of MC2884 directly reduced the expression of EZH2 and induced apoptosis with implications that MC2884 reduced both methylation and acetylation at H3K27. The potential of this inhibitor to shape the leukemic epigenome and upregulate apoptosis has important implications for APL treatment (Singh et al., 2018).

Disruptor of telomeric silencing 1 (DOT1L) is a histone methyltransferase that targets the histone H3 lysine 79 position, or H3K79 (Feng et al., 2002). Uniquely, DOT1L does not bind in the same way as SET domain methyltransferase proteins tend to (Feng et al., 2002), and the unique AdoMet-binding method of DOT1L makes it an important target for therapeutic treatments (Sawada et al., 2004). Notably, differential degrees of methylation of the histone H3 lysine 79 position lead to varying cellular responses. H3K79 mono-methylation (H3K79me1) and di-methylation (H3K79me2) have been correlated with transcriptional activation (Zhang et al., 2004; Morillon et al., 2005), and analyses in a *Saccharomyces cerevisiae* yeast model have shown differential levels of H3K79me2 at various stages of the cell cycle (Schulze et al., 2009), indicating a potential regulatory role of H3K79 methylation in the cell cycle. Though there are some genes for which tri-methylation of H3K79 (H3K79me3) has an activating effect, H3K79me3 has been generally correlated with gene repression in human T cells.

DOT1L has additionally been implicated in oncogenesis for multiple leukemias, including mixed lineage leukemia (MLL), acute lymphoblastic leukemia (ALL), and acute myeloid leukemia (AML) (McLean et al., 2014). In particular, high levels of H3K79 methylation, indicative of high levels of DOT1L activity, were found in an analysis of MLL (Deshpande et al., 2013). Human DOT1L further interacts with AF10, an MLL-fusion partner, resulting in the upregulation of multiple leukemia-relevant genes, including *HOXA9* and *HOXA7* (Okada et al., 2005).

Due to the suspected role of DOT1L in oncogenesis, inhibition of this histone methyltransferase has been of interest. While multiple DOT1L inhibitors such as EPZ04777 (Daigle et al., 2011) and SGC0946 (Yu et al., 2012) have shown ability to inhibit the histone methyltransferase, they have non-optimal pharmacokinetic properties (Daigle et al., 2011). There has been some evidence that a DOT1L inhibitor such as pinometostat may have clinical efficacy if paired with other inhibitors or drugs (Stein et al., 2018), but further research needs to be conducted to design a robust DOT1L inhibitor that can be used safely for treatment in humans.

There exist additional epigenetic modalities of treatment, including histone demethylase inhibitors (D’Oto et al., 2016; Li et al., 2018), bromodomain-containing protein inhibitors (Zuber et al., 2011; Pérez-Salvia and Esteller, 2017), and mutant isocitrate dehydrogenase inhibitors (DiNardo et al., 2017; Nassereddine et al., 2017). These therapies offer promising further avenues for the epigenetic modulation of acute myeloid leukemia.

## Histone Deacetylase Inhibitors

Histone deacetylase (HDAC) inhibitors are posited to treat cancers by preventing excessive deacetylation of histones and transcription factors, particularly those that regulate tumor-suppressor genes (Garcia-Manero et al., 2008). Alternatively, HDAC inhibitors could function through hyperacetylation that leads either to transcription factor overexpression or to gene acetylation (Glaser et al., 2003). One benefit of using HDAC inhibitors as clinical treatments is that they primarily mediate cancerous cell death and exhibit less cytotoxicity in healthy cells (Xu et al., 2007), thereby making them a more attractive therapeutic agent.

Generally, HDACs deacetylate lysine residues on proteins, including those that regulate cell death and cell proliferation, and are classified into one of four classes based on their similarity to yeast HDACs (Xu et al., 2007). The class I HDACs are HDACs 1, 2, 3, 8, and 11 (Glaser et al., 2003). This class of HDAC is categorized based on its homology to the yeast Rpd3 HDAC (Haberland et al., 2009). Structurally, the most common HDAC inhibitors include a hydroxamic acid or a benzamide zinc-binding group; however, current research has identified potential HDAC inhibitors that can bind zinc without the “pharmacokinetic liability” of the hydroxamic acid or toxicity of benzamide groups (Li and Woster, 2015). HDAC inhibitors can function by inhibiting cells from exiting the G1 stage of the cell cycle, by inducing apoptosis through upregulating death receptors and ligands or inducing the mitochondria driven apoptotic pathway, by causing DNA defects leading to cell death during mitosis, and by causing autophagy, among many possible pathways (Xu et al., 2007).

The drug panobinostat, for example, is an HDAC inhibitor used to treat AML. It functions to inhibit class I HDACs and degrade the oncoprotein AML1/ETO9a, which drives AML disease (Bots et al., 2014). It shows promise for treating leukemia that has an acquired resistance to apoptosis, including in *p53* knockout cells and in cells treated with anti-apoptotic agents (Bots et al., 2014) and which can play a role in controlling the cell cycle (Chiron et al., 2013). Vorinostat is another HDAC inhibitor that shows promise in the treatment of AML. Phase 1 trials of vorinostat combined with cytarabine chemotherapy for patients with relapsed or refractory AML demonstrated positive clinical outcomes: 6 of 17 (35%) patients achieved complete remission, although 5 of those patients later relapsed and died (Mims et al., 2018). Interestingly, concurrent treatment of vorinostat with azacitidine did not improve clinical outcomes in comparison with patients receiving only azacitidine, which could be an indication that vorinostat interferes with cellular uptake of azacitidine (Craddock et al., 2017). In contrast, a JAK2-HDAC1 dual inhibitor showed significant *in vivo* anti-proliferative potential while also working to prevent opportunistic fungal infections, which are a consistent threat to immunocompromised AML patients (Huang et al., 2018). Overall, HDAC inhibitors show strong promise to treat some of the epigenetic bases of AML, although it is necessary to further examine combination therapies to ensure that the drugs do not interfere with each other’s ability to function.

## DNA Methyltransferase Inhibitors

Decitabine (2′-deoxy-5-azacytidine) and azacytidine (5-azacytosine) are cytosine analogues (azanucleosides) that inhibit the function of DNA methyltransferase, an enzyme responsible for catalyzing the methylation of DNA (Jones and Taylor, 1980). As the inhibition of methylation can revert the hypermethylation-induced silencing of tumor-suppressor genes (Mund et al., 2006), the use of DNA methyltransferase inhibitors as therapeutic agents offers promising implications for acute myeloid leukemia and related malignancies.

Decitabine inhibits DNA methylation and suppresses growth in multiple human tumor cell lines by reactivating methylation-silenced tumor suppressors and regulators (Bender et al., 1999). Decitabine has been shown, for example, to demethylate and reactivate hypermethylated versions of the *p51* tumor-suppressor gene, aiding in the restoration of normal cell cycle and function (Daskalakis et al., 2002). Decitabine has also been shown to upregulate BASP1, which is epigenetically silenced (*via* methylation of the BASP1-promoter region) in acute myeloid leukemias characterized by the t(8;21) translocation (Zhou et al., 2018). In a clinical analysis, continuous administration of decitabine resulted in genome-wide hypomethylation in patients with refractory solid tumors, though this hypomethylation was impermanent, with genomic methylation levels returning to pre-treatment baseline levels within 4–5 weeks (Samlowski et al., 2005).

The incorporation of azanucleosides into DNA and the associated inhibition of DNA methylation involve a complex set of biochemical mechanisms involving cellular uptake, intracellular metabolism, and azanucleoside incorporation. Though the precise mechanism of azanucleoside cellular uptake is uncertain, nucleoside transporters (Hubeek et al., 2005) and the equilibrative transporter ENT-1 (Huang et al., 2004) have been implicated in the process. Following cellular uptake, azanucleosides are metabolically activated and modified into 5-aza-2′-deoxycytidine-5′-triphosphate. The nucleoside is phosphorylated (ATP-dependent) into the monophosporylated nucleotide. The metabolic activation of both azacytidine and decitabine is catalyzed by enzymes specific to each compound (Momparler and Derse, 1979); deoxycytidine kinase has, for example, been implicated in the metabolic activation of decitabine (Stegmann et al., 1995).

Following their metabolic activation, azanucleosides are incorporated into DNA during DNA replication as replacements for cytosine. This leads to the formation of azacytosine-guanine dinucleotides, which are recognized by DNA methyltransferase. After recognition, methylation is initiated *via* a nucleophilic attack, resulting in the formation of a covalent bond between the carbon-6 atom on the azacytosine ring and the DNA methyltransferase enzyme. Unlike in the case of the interaction between regular cytosine and DNA methyltransferase, where a β-elimination occurs through the carbon-5 atom on the cytosine ring, azacytosine (where the carbon-5 is replaced by a nitrogen) and DNA methyltransferase remain covalently bound to one another until the methyltransferase enzyme is eventually degraded or removed (Santi et al., 1984). This mechanism serves to inhibit the function of the DNA methyltransferase, preventing methylation.

While some enzymes aid the process of azanucleoside activation and incorporation, there exist enzymes (e.g., cytidine deaminase), which have been implicated in the inhibition of this metabolic process (Chabot et al., 1983; Chuang et al., 2010). Thus, it is important to consider ways of countering these inhibitors and enhancing azanucleoside stability in order to optimize azanucleoside therapy. Indeed, the chemical stability of azanucleosides is an important consideration for determining the clinical viability of these compounds. In particular, the plasma stability of azanucleoside-class compounds is an important consideration for clinical contexts. S110, a dinucleotide that contains 5-aza-2′-deoxycytidine and a deoxyguanosine, is comparatively resistant to deamination by cytidine deaminase (Chuang et al., 2010) while still retaining a comparable ability to inhibit methylation (Yoo et al., 2007). Promisingly, azanucleosides further have considerable stability at both room and physiological temperatures, with this class of compounds still retaining efficiency in inhibiting genomic cytosine methylation subsequent to storage at room temperature (Stresemann and Lyko, 2008).

Though demethylation-based approaches are promising avenues of therapy for acute myeloid leukemia and similar malignancies, it is important to note that there are concerns associated with excessive hypomethylation. Hypomethylation has been shown in mouse models to lead to tumors *via* the genetic amplification (Gaudet et al., 2003) and insertional activation of oncogenic loci (Howard et al., 2008). Overall, while azanucleoside therapy is currently one of the most advanced epigenetic cancer therapies with a significant therapeutic potential in acute myeloid leukemia, further study is needed to better understand the molecular mechanisms and safety considerations of such therapies. Moreover, new combined CRISPR systems that use specific guide RNAs to selectively target specific loci for methylation or de-methylation can also turn the genome-wide therapy into one with more focused impact (Afshinnekoo and Mason, 2016).

## Menin-MLL Inhibitors

Patients with *de novo* AML are found to have a chromosomal translocation of the mixed lineage leukemia gene located at chromosome band 11q23 (Cierpicki and Grembecka, 2014), a chromosomal translocation that results in the fusion of the MLL gene with one of over 60 different protein partners (Prange et al., 2017), with the AF9 protein being a common fusion partner in mixed lineage leukemia (Prange et al., 2017).

The disruption of the MLL gene caused by the gene fusion leads to the upregulation of *HOXA9* and *MEIS1* gene expression (Zeisig et al., 2003), the upregulation of which ultimately results in the inhibition of hematopoietic differentiation and the enhancement of cellular proliferation, resulting in acute leukemia (Zeisig et al., 2003). While chemotherapy is the standard treatment for this type of leukemia, the event-free survival is a comparatively low 50% for both pediatric and adult patients (Neff and Armstrong, 2013), necessitating novel treatment approaches.

MLL fusion proteins are dependent on interactions with menin, a protein encoded by the *MEN1* gene, for their oncogenic function (Yokoyama et al., 2005). Menin binds to N-terminus of MLL in the MLL fusion protein (Caslini et al., 2007) and recruits the MLL fusion protein to activate genes, including *HOXA9* (Chen et al., 2006).

Inhibiting the ability of menin to bind to MLL fusion proteins has been shown to inhibit cellular proliferation and induce cellular differentiation (Grembecka et al., 2012). Both the direct genetic disruption of the menin-MLL fusion protein interaction (Yokoyama et al., 2005) and the use of small-molecule inhibitors to block the menin-MLL protein interaction (Grembecka et al., 2012; He et al., 2014) induced cellular differentiation and reduced cellular proliferation *in vitro* (Yokoyama et al., 2005; Grembecka et al., 2012; He et al., 2014).

Borkin et al. characterized two additional small-molecule inhibitors – MI-463 and MI-503 – for *in vivo* use (Borkin et al., 2015). Introduction of MI-503 into a leukemic mouse model resulted in more than an 80% reduction in MV4;11 tumor volume in two mice, and treatment with MI-463 or MI-503 resulted in the median survival of MLL-AF9 leukemic mice increasing by ~70% and ~45%, respectively (Borkin et al., 2015). Further, MI-503 was noted to be metabolically stable, orally bioavailable, and lacking any noticeable hepatotoxic or nephrotoxic effects after prolonged, 38-day treatment in mice (Borkin et al., 2015), indicating therapeutic promise for the *in vivo* applications of this menin-MLL inhibitor.

## Bromodomain-Containing Protein Inhibitors

Bromodomains (BRDs) are protein domains involved in the regulation of gene expression *via* recruitment of various molecular partners. BRDs recognize acetylated lysine residues, such as those found on histone tails, and recruit chromatin-modifying enzymes such as histone acetyltransferases (HATs) and histone deacetylases (HDACs) along with other transcriptional machinery to specific sites in the chromatin (Pérez-Salvia and Esteller, 2017).

The bromodomain and extra terminal (BET) protein family includes the proteins BRD2, BRD3, BRD4, BRD9, and BRDT (Pérez-Salvia and Esteller, 2017). BET proteins have been implicated in the regulation of some cancer-related genes (Delmore et al., 2011): for example, BRD4 recruits the positive transcription elongation factor complex (P-TEFb) to acetylated chromatin, leading to the transcriptional initiation and elongation of genes controlling cell proliferation (Yang et al., 2005); and BRD9 facilitates maintenance of the leukemic phenotype (Bakshi et al., 2010; Shi et al., 2013) as a component of the SWItch/Sucrose Non-Fermentable (SWI-SNF) chromatin-remodeling complex (Hohmann et al., 2016).

Delmore et al. demonstrated the use of a small-molecule inhibitor, JQ1, to inhibit BET proteins as regulatory factors for *c-Myc* (Delmore et al., 2011), which has been implicated in the pathogenesis of many cancers (Miller et al., 2012), including acute myeloid leukemia, in which *c-Myc* tends to be upregulated (Luo, 2005). The inhibition of BET by JQ1 has been shown to downregulate *Myc* transcription and, ultimately, *Myc*-dependent target genes, resulting in an overall reduction of cellular proliferation (Delmore et al., 2011).

Additional BET inhibitors have also been characterized. OTX015 (MK-8628) – a BRD2, BRD3, and BRD4 inhibitor – was demonstrated to have a stronger anti-proliferative effect than JQ1 (Berenguer-Daizé et al., 2016). In a similar manner to JQ1, OTX015 results in cell growth inhibition and apoptosis in acute leukemia cell lines (Coudé et al., 2015), and this inhibitor is currently undergoing clinical trials for AML treatment (Dombret et al., 2014).

Additionally, Hohmann et al. demonstrated that the BRD9 domain of SWI-SNF facilitates the leukemogenic ability of this chromatin-remodeling complex by facilitating the *Myc* transcription, the induction of cellular proliferation, and the inhibition of hematopoietic differentiation (Hohmann et al., 2016) that characterizes acute myeloid leukemia. The authors of this work further demonstrated the use of small-molecule BRD9 inhibitors – particularly, BI-7273 – that were effective in curbing cellular proliferation in AML cell lines (Hohmann et al., 2016). Martin et al. described another BRD9 inhibitor – BI-9564 – with an improved pharmacokinetic profile relative to that of BI-7273 (Martin et al., 2016).

## Mutant Isocitrate Dehydrogenase (IDH1) Inhibitors

Approximately 6–10% of patients with acute myeloid leukemia (AML) have mutations in the isocitrate dehydrogenase 1 (IDH1) gene (DiNardo et al., 2018). IDH1 is involved in the regulation of cellular metabolism, specifically lipid metabolism given the ability of IDH1 to produce cytoplasmic NADPH (Koh et al., 2004) and in glucose sensing (Joseph et al., 2006). Wild-type isocitrate dehydrogenases (IDH) are enzymes that catalyze the oxidative decarboxylation of isocitrate to α-ketoglutarate (Cairns and Mak, 2013), and it has been suggested that the IDH1 Arg^132^ mutation causes a change in enzyme function, leading to the reduction of α-ketoglutarate to R(−)-2-hydroxyglutarate (2HG) (Dang et al., 2009). This excess of R(−)-2-hydroxyglutarate leads to an increase in cellular proliferation and impairs cellular differentiation (Losman et al., 2013).

Furthermore, 2HG has been shown to inhibit histone demethylation (Xu et al., 2011), a problematic effect given that histone methylation can result in the silencing of tumor-suppressor genes (Agrawal et al., 2007). Thus, researchers are exploring 2HG inhibition to reverse this hypomethylation, for instance, Li et al. demonstrated the use of AGI-5198, an IDH1 inhibitor, to inhibit 2HG production (Li et al., 2015). Treatment with AGI-5198 of cells containing mutated IDH1 indicated anti-tumoral activity and induced apoptosis (Li et al., 2015), suggesting potential clinical promise.

In 2018, the Food and Drug Administration (FDA) approved ivosidenib (Tibsovo®), an inhibitor of IDH1/2HG production, for adult AML patients with the IDH1 mutation (Dhillon, 2018), following clinical trials, which indicated that ivosidenib induced remissions and improved patient outcomes (DiNardo et al., 2018).

## Histone Demethylase Inhibitors

Lysine-specific demethylase 1 (LSD1) is an enzyme responsible for the demethylation of histone H3 lysine 4, playing an important role in epigenetic regulation (Shi et al., 2004). LSD1 expression is enriched in multiple cancer cell types (Hayami et al., 2010), and LSD1 inhibition has been shown to induce cellular differentiation and inhibit cellular proliferation (Schulte et al., 2009).

In a mixed lineage leukemia mouse model, mice treated with LSD1 inhibitor showed substantial decreases in leukemic cell proliferation, but the treated mice presented with greater levels of anemia and thrombocytopenia compared to vehicle-treated control mice (Harris et al., 2012). Thus, many of the mice treated with LSD1 inhibitor died of anemia rather than leukemia, though this problem could be addressed in humans *via* adjustments to dosage and *via* blood transfusions (Harris et al., 2012). Though LSD1 presents as a potential therapeutic target in mixed lineage leukemias, additional considerations are necessary before usage of LSD1 inhibitors in human treatment.

It has been suggested that the pairing of an LSD1 inhibitor with other therapeutic agents may be more effective than the use of the LSD1 inhibitor alone. Schenk et al. demonstrated in mouse models of AML that combination therapy – all-trans retinoic acid (ATRA), which enhances cellular differentiation, in conjunction with tranylcypromine (an LSD1-inhibitor) – decreased tumor burden more substantially than the use of either agent alone (Schenk et al., 2012). Furthermore, their work suggests that LSD1 inhibits the pro-differentiative function of ATRA, and thus the inhibition of LSD1 *via* tranylcypromine allowed for ATRA to induce cellular differentiation to a more prominent extent (Schenk et al., 2012). This, along with other results from other studies (Ishikawa et al., 2017), points to the therapeutic potential in AML of combination therapy utilizing LSD1 inhibitors.

## The Impact of Heterogeneity

Cellular heterogeneity is increasingly implicated in oncogenesis. Tumors evolve clonally through a series of stepwise, somatic mutations (Nowell, 1976). Like most cancers, acute myeloid leukemia often presents with intratumoral genetic and epigenetic heterogeneity, and it has been suggested that this heterogeneity is what leads to the high rate of relapse among patients post-therapy. However, relative to other cancers, AML has a comparatively low level of genetic heterogeneity (The Cancer Genome Atlas Research Network, 2013), suggesting that epigenetic heterogeneity is of primary importance (Li et al., 2016a,b), as well as other regulators such as non-coding RNAs and miRNAs (Hu et al., 2015). Here, we discuss epigenetic heterogeneity in acute myeloid leukemia.

## Heterogeneity and Cancer Cell Population Fitness

Like in other cancers, heterogeneity in acute myeloid leukemia is likely maintained due to the increase in fitness associated with a heterogeneous population. In an environment with fluctuating selective pressures (such as one exposed to therapeutic drugs and radiation), it is beneficial for the population to employ a bet-hedging strategy (Brutovsky and Horváth, 2012). Although therapeutic agents (chemotherapy, radiation, etc.) are designed to eradicate cancerous cells, they are also a source of selective pressure in which therapy-resistant subclones are selected for (Turner and Reis-Filho, 2012; Greaves and Maley, 2012). In this way, cancer evolution can be thought of as a traditional Darwinian process in which less fit subclones are eliminated from the population by therapeutic agents (i.e., bet-hedging). More recently, there has been evidence that bet-hedging is not the only reason why heterogeneity is maintained in a population: increased epigenetic heritability for mutations that hasten mean cell division time and increased heterogeneity have been found to be positively correlated with mean population fitness in yeast (Cerulus et al., 2016).

## Next Generation Sequencing and AML Heterogeneity

Recent research coupled with the development of next generation single-cell sequencing has advanced the characterization of AML and elucidated both the true complexity of and the genetic heterogeneity associated with the disease. Many genes, such as *FLT3* and *NPM1*, display both homogeneity and heterogeneity in AML populations and appear to be undergoing convergent evolution (Paguirigan et al., 2015). More difficult to ascertain is the level of epigenetic heterogeneity in a sample, as epigenetic modifications are often dynamic. The study of epigenetic heterogeneity in AML is further complicated by the detection limit of sequencing (Chhangawala et al., 2015) and the high level of temporal and environmental phenotypic plasticity of AML epigenetic changes (Li et al., 2016a,b).

Epigenetic heterogeneity in acute myeloid leukemia is also present in differential chromatin landscapes and gene expression profiles. Through the comparison of two AML subtypes with two different translocations [(t8;21), in which the RUNX1-binding domain is fused to the ETO regulator, and t(3;21), in which the RUNX1-binding domain is fused to the EVI1 regulator], Loke et al. determined that these two subtypes each displayed a unique epigenetic landscape and gene expression profile. Each type had a different transcriptional network, which likely explains the differing clinical outcomes for the two types (Loke et al., 2017; Agirre et al., 2018). Clearly, it is necessary to not only consider genetic heterogeneity but also consider epigenetic heterogeneity to comprehensively characterize acute myeloid leukemia pathophysiology.

## Genetic Heterogeneity

Interestingly, epiallele diversity may develop before AML and is enhanced by cooperation between somatic mutations (The Cancer Genome Atlas Research Network, 2013; Li et al., 2017). For example, mutational cooperativity between *FLT3* mutations and *TET2* mutations has been shown to confer a gain-of-function DNA methylation, leading to differentiated gene expression (Shih et al., 2015). In acute myeloid leukemia, somatic mutations are most often classified as nonsynonymous mutations in genes relevant for pathogenesis. Some of these changes influence epigenetic modifications, such as mutations in DNA-methylation genes and chromatin-modifying genes (The Cancer Genome Atlas Research Network, 2013). However, there is debate as to whether there is an association between the degree of epigenetic heterogeneity and the somatic mutational burden in epigenetic modifier genes (Li et al., 2016b) or not (Li et al., 2016a,b).

Epiallele diversity is inversely correlated with clinical outcome in acute myeloid leukemia, and more complex epialleles are associated with higher-risk AMLs (Li et al., 2016a,b; Li et al., 2017). More heterogeneous epialleles and higher epigenetic burden have been correlated with increased rate of relapse (Garrett-Bakelman et al., 2015). For example, children with the common t(8;21)(q22;q22)/*RUNX1-RUNX1T1* mutation are more likely to relapse if they display heterogeneous DNA methylation at the time of diagnosis. Patients who relapsed had a unique epigenetic signature that led to aberrant activation of several cell-to-cell adhesion and cell-motility pathways, which likely contributed to their relapse (Zampini et al., 2018). This relationship is unsurprising, as epigenetic modification is a core component of plasticity, and increased plasticity usually confers increased fitness. A significant increase in the level of epigenetic heterogeneity precedes a significant change in somatic mutational burden, which also likely contributes to increased tumoral fitness and to therapeutic resistance upon relapse (Garrett-Bakelman et al., 2015).

## Heterogeneity as a Driving Force

Acute myeloid leukemia can be generally characterized as “driven” by one of two driving factors in disease progression: higher epigenetic heterogeneity and lower mutational burden (epigenetically driven), and lesser epigenetic heterogeneity and higher mutational burden (genetically driven). The genetically driven cluster increases in epigenetic heterogeneity as the disease progresses (Li et al., 2016a,b). As relapse is often followed by mortality in acute myeloid leukemia, relapse states are important considerations for AML prognosis and progression. While the bulk of the research regarding the effect of heterogeneity on treatment efficacy, relapse occurrence, and disease prognosis is focused on genetic heterogeneity (Ding et al., 2012; Fisher et al., 2013; Hackl et al., 2017), several models have been developed that can predict prognosis and clinical outcome from methylation levels (Bullinger et al., 2009).

## Targeted Epigenetic Therapies and the Heterogeneity Problem

Mutations in the isocitrate dehydrogenase 2 (*IDH2*) gene, which commonly lead to hematopoietic differentiation arrest, are a progenitor to AML. Enasidenib is a selective small-molecule inhibitor of *IDH2*; mutant *IDH2* inhibition promotes leukemic cell differentiation. Many patients treated with enasidenib relapse due to selection for resistant clones and subsequent clonal evolution. Quek et al. recently used enasidenib as a model pathway to develop a method to reveal how cancerous subclones respond to therapeutic agents *via* clonal mapping throughout disease progression (Quek et al., 2018). Additionally, combined therapy using various IDH1 inhibitors has shown promise in resetting the epigenetic landscape to a baseline hematopoietic state (Shih et al., 2017).

However, there is evidence that epigenetic-targeted therapies can help to decrease epiallele diversity in acute myeloid leukemia. This is a likely reason for the efficacy of epigenetic treatments in AML, as overall AML epigenetic diversity decreases, so does overall AML fitness (Li et al., 2017). There exists difficulty in developing a method to target all possible subclones, so as to eliminate the possibility for resistance *via* clonal evolution (Roboz, 2014). This indicates that continued patient monitoring will likely be required, across both the genetic and epigenetic dimensions, to gauge the impact of therapy and optimize changes in patient treatments.

## Future Directions

Acute myeloid leukemia is a complex disease characterized not only by significant genetic mutation but also by deleterious epigenetic differentiation, which has motivated the development of therapies that target epigenetic modifications in AML. However, as these epigenetic modifications are heritable and highly heterogeneous, acute myeloid leukemia undergoes significant clonal evolution, culminating in therapy resistance and eventual relapse. New animal models are being explored that can begin to tease out aspects of these diseases, such as in canine and primates (Pipes et al., 2013; Peng et al., 2015), and these can accelerate testing of the epigenetic hypotheses described here. Also, new sequencing technologies can improve both the accuracy and speed of characterizing AML, such as those that leverage hybrid assembly approaches (e.g., short read and long read) to study the complexity of the genome (Rosenfeld et al., 2016) and rapid, real-time sequencing methods that work in almost any environment (McIntyre et al., 2016; Castro-Wallace et al., 2017) can expand access to diagnostic approaches in NGS. Finally, efforts to explore and describe the heterogeneous epigenetic landscape of acute myeloid leukemia (e.g., *via* single-cell next generation sequencing) offer promising avenues for the optimization of AML therapies by taking the characteristic heterogeneity of acute myeloid leukemia into account, thereby improving outcomes for this malignancy.

## Author Contributions

SG, CH, and MK equally authored the significant portions of the work, and SG and CH directed the development of the manuscript. AS, YJ, and EA contributed to individual sections of the work. EA provided feedback. CM contributed to various sections throughout the manuscript and provided guidance and feedback regarding the overall direction of the work.

### Conflict of Interest Statement

CM is a cofounder and a board member of Biotia and Onegevity Health, as well as an advisor or a compensated speaker of Abbvie, Acuamark Diagnostics, ArcBio, BioRad, DNA Genotek, Genialis, Genpro, Karius, Illumina, New England Biolabs, QIAGEN, Whole Biome, and Zymo Research.

The remaining authors declare that the research was conducted in the absence of any commercial or financial relationships that could be construed as a potential conflict of interest.
